# Biocontrol Microneedle Patch: A Promising Agent for Protecting Citrus Fruits from Postharvest Infection

**DOI:** 10.3390/pharmaceutics15041219

**Published:** 2023-04-11

**Authors:** Ling Jiang, Huan Huang, Xingyu Shi, Jian Wu, Juexian Ye, Qian Xu, Shaobin Fang, Chuanbin Wu, Rui Luo, Chao Lu, Daojun Liu

**Affiliations:** 1 Department of Pharmacy, Shantou University Medical College, Shantou 515041, China; 2 College of Pharmacy, Jinan University, Guangzhou 511436, China; 3 Shantou Central Hospital, Shantou 515041, China; 4 Guangdong Provincial Key Laboratory of Infectious Diseases and Molecular Immunopathology, Shantou University Medical College, Shantou 515041, China; 5 The Second Affiliated Hospital of Shantou University Medical College, Shantou 515000, China

**Keywords:** biocontrol microneedle patch, antifungal agent, ε-poly-lysine, citrus fruit, postharvest infection

## Abstract

With increasing human awareness of food safety, the replacement of highly toxic pesticides with biocompatible antimicrobials has become a trend. This study proposes a biocontrol microneedle (BMN) to expand the application of the food-grade preservative epsilon-poly-L-lysine (ε-PL) in fruit preservatives by utilizing a dissolving microneedle system. The macromolecular polymer ε-PL not only possesses broad-spectrum antimicrobial activity but also exhibits good mechanical properties. With the addition of a small amount of polyvinyl alcohol, the mechanical strength of the ε-PL-based microneedle patch could be further improved to achieve an enhanced failure force of needles at 1.6 N/needle and induce an approximately 96% insertion rate in citrus fruit pericarps. An ex vivo insertion test revealed that the microneedle tips could be effectively inserted into the citrus fruit pericarp, rapidly dissolve within 3 min, and produce inconspicuous needle holes. Moreover, the high drug loading capacity of BMN was observed to reach approximately 1890 μg/patch, which is essential for enhancing the concentration-dependent antifungal activity of ε-PL. The drug distribution study has confirmed the feasibility of mediating the local diffusion of EPL in the pericarp through BMN. Therefore, BMN has great potential to reduce the incidence of invasive fungal infections in local areas of citrus fruit pericarp.

## 1. Introduction

Fruits (e.g., citrus, grapes, and apples) have high economic and nutritional values but high nutrient and water contents, which make them susceptible to postharvest mold infections [1,2,3]. Numerous types of pathogens exist that induce fruit diseases in nature, including *Penicillium digitatum*, *Penicillium italicum*, *Penicillium expansum*, *Botrytis cinerea*, and *Alternaria alternata* [4,5,6,7,8]. Therefore, the application of broad-spectrum antifungals is important for the postharvest disease control of fruits.

Artificial fungicides, including thiabendazole, sodium ortho-phenylphenol, and imazalil, have been extensively used to inhibit the spread of postharvest fruit diseases. However, residues of these chemical fungicides are usually toxic and carcinogenic and may induce chronic or acute toxicity in humans [9,10,11]. In contrast, epsilon-poly-L-lysine (ε-PL), which typically consists of 25–40 L-lysine residues, has been approved by the US Food and Drug Administration (FDA) as a food-grade preservative because of its broad-spectrum activity, biodegradability, and biocompatibility [12]. Recently, ε-PL has been widely used in the preservation of instant rice, cooked noodles, cooked vegetables, seafood, sauces, soy sauce, and crackers [13,14]. However, fruit cuticles primarily composed of polymer cutin and cuticular wax provide effective surface barriers, which are not conducive to the penetration of polar macromolecules such as ε-PL [15,16]. The low percutaneous efficiency of ε-PL may severely hinder its inhibition of pathogens colonizing the deep pericarp. Therefore, the application of ε-PL in controlling the postharvest decay of fruits is still limited and remains only in the field of basic research.

The microneedle patch is a novel topical drug delivery system in which the drug is incorporated into tiny needles at the micron level and the needles are attached to a base-supporting patch. Drugs can pass through the tissue barrier via needle tips by pressing the microneedle patch onto the tissue surface with significantly improved transdermal delivery efficiency [17,18]. Owing to the recent increase in the level of mass production, the reduction in production costs, and the advantages of its universality, microneedle patches have been developed for skin disease treatment [19,20,21,22], blood alcohol and glucose detection [23,24,25], and vaccination [26,27,28,29].

**Scheme 1:**
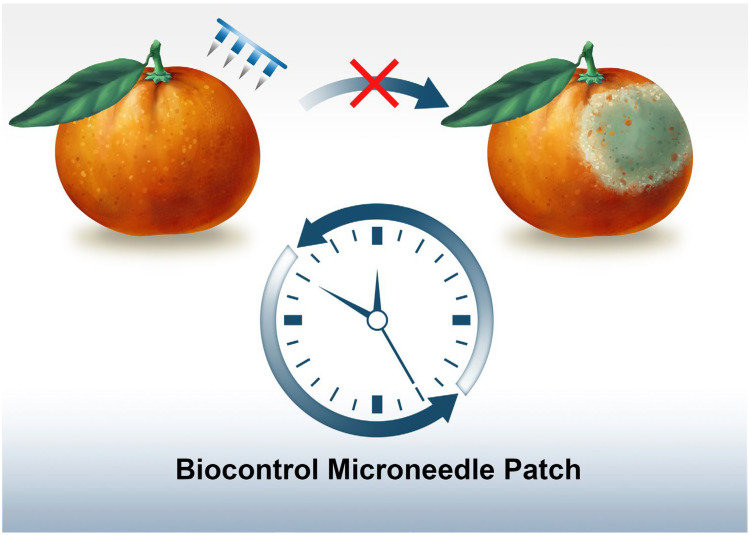
Schematic illustration of using BMN to protect fruits from infection.

Considering the above-mentioned advantages of microneedles, this study proposes the construction of a biocontrol microneedle-array patch (BMN) based on the food-grade preservative ε-PL to explore its potential application in controlling the decay of fruits, particularly citrus fruits (Scheme 1). An ex vivo insertion test of various microneedles in pericarp was first carried out to screen the optimal microneedle formulation. Subsequently, the drug loading amount, microneedle mechanics, and insertion ability of the ε-PL-based BMN were characterized. To elucidate the mode of action after microneedle administration, the dissolution and diffusion behavior of BMN have been investigated. Finally, cytotoxicity experiments were conducted to demonstrate the safety of BMN. This ε-PL-based microneedle patch is expected to exhibit the following advantages: (1) In addition to serving as a broad-spectrum antimicrobial, ε-PL with a molecular weight of 3.2~4.4 kDa possesses the mechanical properties of polymeric materials [30], which can improve the drug loading capacity by reducing the amount of microneedle tip excipients. (2) Using the dissolving microneedle patch system, ε-PL can efficiently penetrate into the citrus pericarp to exert better mold prevention and control. (3) ε-PL can be degraded into natural amino acids required by the human body in the gastrointestinal tract after oral administration, and its safety is much higher than that of traditional pesticides [31]. (4) The micron-level pores produced by microneedle piercing will naturally shrink with the evaporation of water on the pericarp surface, which is conducive to reducing the entry of microorganisms into the citrus through the pores, while ensuring the aesthetics of the fruit surfaces. (5) The improvements of large-scale production, the reduction in production costs, and the development of potential advertising uses in recent years are expected to reduce the cost of antimicrobial microneedles in fruit preservation.

## 2. Materials and Methods

### 2.1. Materials

Polyvinyl alcohol (PVA, 103) and trypan blue were obtained from Aladdin (Shanghai, China). Polyvinyl pyrrolidone (PVP K90) were kindly donated by BASF (Ludwigshafen, Germany). ε-PL was purchased from Binafo Biology Co., Ltd. (Zhengzhou, China). L-lysine hydrochloride and 4% paraformaldehyde were purchased from Sigma-Aldrich (Shanghai, China). An adhesive label was purchased from Deli (Ningbo, China). Sulfo-Cyanine-7 NHS ester (Cy7) was purchased from Xi’an Qianghua Biological Technology Co., Ltd. (Xi’an, China). All the other solvents and materials used were of analytical grade.

### 2.2. Fabrication and Imaging Study of Various Microneedles

BMN were prepared by a multi-step centrifugation method using female polydimethylsiloxane (PDMS) molds fabricated by replica molding from a microneedle array template. First, a template of a microneedle patch with a 12 × 12 array of tetragonal pyramidal needles was fabricated using computer-aided design and computer-aided manufacturing cutting operations with brass as the raw material.

The master molds were placed in anhydrous ethanol and cleaned via ultrasonication. Then, a 10:1 mixture of polydimethylsiloxane (PDMS) monomer and curing agent (Dow Corning Slygard 184) was poured into the mold, degassed under vacuum for 30 min, and cured at 80 °C for 2 h. After cooling to room temperature, a PDMS mold with a morphology complementary to that of the master mold was obtained.

Subsequently, ε-PL was mixed with ultrapure water at a ratio of 1:1.8 (*w*/*v*) and stirred until completely dissolved. PVA solution was obtained by mixing PVA with ultrapure water at a ratio of 1:1.8 (*w*/*v*) and water bath at 90 °C for 2 h. Needle tip solutions containing different amounts of ε-PL were obtained by mixing ε-PL solution with PVA solution at the mass ratios of 1:9, 3:7, 5:5, 7:3, and 9:1, respectively. After a water bath at 65 °C for 1 h, 250 μL of the needle tip solution was added to the PDMS molds and centrifuged at 3500× *g* at 20–30 °C for 10 min. After removing excess needle tip solution from the molds, the drug-containing needle tips were centrifuged at 3500× *g* at 20–30 °C for 10 min to allow the molds to be filled and properly concentrated. Further, 26.2% ethanolic solution of PVP K90 was added to the PDMS molds and centrifuged at 3500× *g* for 45 min at 0–10 °C. Finally, the BMN were gently separated from the PDMS molds by drying in a desiccator for 72 h at room temperature. To evaluate the distribution of ε-PL in the tip, microneedles with a PVP layer stained with trypan blue were prepared using a method similar to that described above, except that an appropriate amount of trypan blue dye was dissolved in the PVP K90 ethanol solution.

A digital camera and inverted microscope (Eclipse Ts2, Nikon Corporation, Japan) were used to observe the morphology of the different microneedles. In addition, the newly prepared microneedles were sputter-coated and imaged using a SU8010 scanning electron microscope (SEM, Hitachi, Tokyo, Japan).

### 2.3. Fruit Treatment

Organically farmed lemons and Satsuma mandarins were purchased from a local citrus farm in Sichuan and Guangxi provinces in China, respectively. Valencia oranges and green pomelos imported from South Africa and Thailand, respectively, were purchased in a local supermarket. All citrus fruits of the four species without any apparent surface damage and infection were chosen, washed with deionized water, and drained until dryness.

### 2.4. An Ex Vivo Insertion Test of Various Microneedles in Pericarp

To evaluate the insertion ability of the different dissolving microneedles in citrus fruits, different microneedles were inserted into the pericarp of freshly separated citrus fruits for 5 min. After removing the microneedles, a 4% (*w*/*v*) solution of trypan blue was added dropwise to the pericarp, where the microneedles were inserted for 2 min. The excess dye solution was washed with distilled water. Stained pores on the pericarp surface were observed and photographed using a digital camera. The efficiency of microneedle tip insertion into the pericarp was calculated as follows:

Insertion efficacy (%) = N_p_/N_n_ × 100% (1)

where N_p_ and N_n_ are the number of stained pores on the pericarp surface and needle tips on the microneedles, respectively.

### 2.5. Determination of the Thickness and Water Content of Various Pericarp

To evaluate the pericarp thickness of the different fruits, pericarp discs with a diameter of 3 cm were cut and separated from the equatorial plane of the different citrus fruits, and the pericarp thickness was measured using Vernier calipers.

To evaluate the moisture content of the different pericarps, freshly obtained pericarps were weighed and dried in an oven (Boxun, Shanghai, China) at 70 °C until they reached a constant weight. The water content of the pericarp was then determined and calculated as follows:

Water content (%) = (W_0_ − W)/W_0_ × 100% (2)

where W_0_ and W are the pericarp weights before and after drying, respectively.

### 2.6. Drug Loading Amount of BMN

To evaluate the loading amount of ε-PL in BMN, the needle part of the BMN was carefully separated using a scalpel blade and dissolved in H_2_O. Subsequently, the ε-PL content of the samples was analyzed by high-performance liquid chromatography (HPLC, Agilent technologies, Santa Clara, CA, USA) using a Waters X-Bridge TM C18 analytical column (4.6 mm × 150 mm, 3.5 μm). The samples were eluted with a linear gradient of water-acetonitrile with 0.1% trifluoroacetic acid at a flow rate of 1 mL/min and were detected at 215 nm.

### 2.7. Microneedle Mechanics

The mechanical strength of the various microneedles was measured using a texture analyzer (TA-XT Plus, Stable Micro Systems, Godalming, UK) [32]. Briefly, the microneedle was placed on the surface of the metallic platform of the texture analyzer. The probe was programmed to move down toward the microneedles patches at a rate of 0.1 mm/s until mechanical fracture occurred. In this study, force was applied parallel to the microneedle axis. Stress versus strain curves were obtained by measuring the force and displacement.

### 2.8. Pericarp Morphology after Microneedle Insertion

To further observe the effect of microneedle insertion on pericarp morphology, fresh pericarps of Satsuma mandarins were obtained. The microneedles were inserted into the surface of the pericarp using thumb pressure for 2 min. After removing the microneedle patches, the pericarp was dried naturally in the air for 30 min and then fixed in 4% paraformaldehyde. Subsequently, pericarp sections were prepared and stained with hematoxylin and eosin (H&E, Beyotime, Shanghai, China) for histopathological observation.

### 2.9. Dissolution Rate of Microneedle Tip in Pericarp

Fresh pericarps of Satsuma mandarin were harvested to investigate the dissolution rate of the microneedle tip in the pericarp. Briefly, microneedles were inserted into the pericarp surface using thumb pressure. At pre-set time points (1, 3, 5, 10, 15, and 30 min), the microneedles were removed and the tip of the microneedle was imaged using an inverted microscope system (Eclipse Ts2, Nikon, Tokyo, Japan).

### 2.10. Study of Drug Distribution after Microneedle Administration

To study the distribution of ε-PL in intact citrus and isolated pericarp, Cy7 was immobilized onto ε-PL and then Cy7-labeled BMN was fabricated. Then, the microneedles were, respectively, inserted into the surface of intact citrus and isolated pericarp for 5 min. At predetermined time points, the fluorescence imaging of citrus and isolated pericarp was conducted using an in vivo imaging system (Lumina III, Perkin Elmer, Waltham, MA, USA).

### 2.11. In Vitro Cytotoxicity Assay

The cytotoxicity of needle-tip excipients on human-originated colon epithelial cells (NCM460) was studied using the cell counting kit-8 (CCK-8) method (Dojindo, Kumamoto, Japan) [33,34]. Briefly, cells were inoculated into 96-well plates and incubated overnight at 37 °C in 5% CO_2_. After 24 h, the medium was removed, and 100 μL of fresh medium containing different concentrations of ε-PL/PVA mixture or L-lysine hydrochloride was added. After another 24 h of incubation, the medium was replaced with 100 μL of fresh DMEM containing 10% fetal bovine serum (Gibco, Grand Island, NY, USA), followed by the addition of 10 μL of CCK-8 solution. After 4 h of incubation, relative cell viability was calculated by measuring the absorbance at 450 nm. The cell viability was calculated as follows:

Cell viability (%) = (A_sample_ − A_blank_)/(A_control_ − A_blank_) × 100% (3)

where A_sample_ is the absorbance of wells containing cells, CCK-8 solution, and sample solution; A_blank_ indicates the absorbance of wells containing medium and CCK-8 solution without cells; and A_control_ indicates the absorbance of wells containing cells and CCK-8 solution but not the sample solution.

### 2.12. Fabrication of Microneedles with an Adhesive Label

An adhesive label was used to wrap the microneedle patch and improve its adherence to the fruit surface. The text and pattern describing the product information were printed on the adhesive label using a printer. The label was then cut into the desired shape. After removing the anti-sticking layer of the adhesive label, microneedle patches were attached to the center of the label.

## 3. Results and Discussion

### 3.1. Preparation of Microneedles Loaded with ε-PL

**Figure 1:**
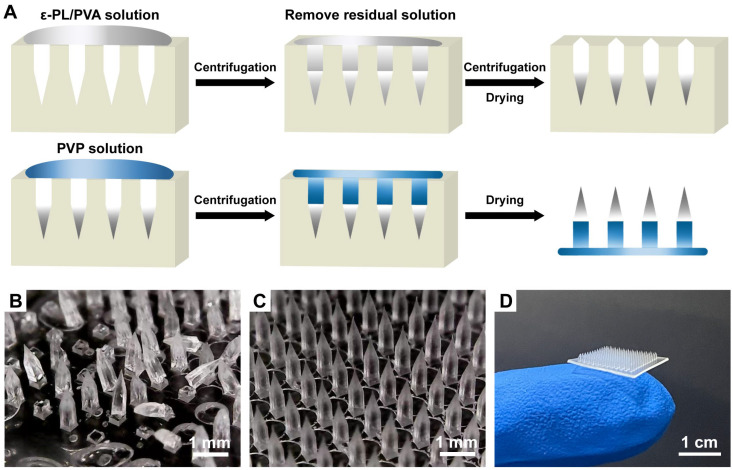
Preparation of ε-PL-loaded microneedles. (**A**) Schematic of the preparation process of ε-PL-loaded microneedles; digital microscope photographs of microneedles with ε-PL to PVA ratio of 10:0 (**B**) and 5:5 (**C**). (**D**) Macroscopic morphology of ε-PL/PVA hybridized microneedles.

We designed a brass template of a microneedle patch with a 12 × 12 array of tetragonal pyramidal needles with a base width of 300 μm and a height of 1200 μm (600 μm pyramidal tip; 600 μm base column). Meanwhile, the tip diameter of the needles was controlled to be no greater than 30 μm, ensuring that the microneedle is sharp enough. PDMS molds fabricated by replica molding from the microneedle array template were used for the fabrication of the ε-PL-loaded microneedles (Figure 1A). As shown in Figure 1B, the microneedles fabricated from pure ε-PL had a high needle breakage rate, and the texture of the tip was extremely brittle. Therefore, we chose PVA, which is a tougher polymer, to improve the tip formability of ε-PL microneedles. The introduction of PVA significantly improved the microneedle shape, resulting in microneedle patches with almost no needle breakage (Figure 1C,D).

### 3.2. Pericarp Insertion Performance and Optimization of Various Microneedles

**Figure 2:**
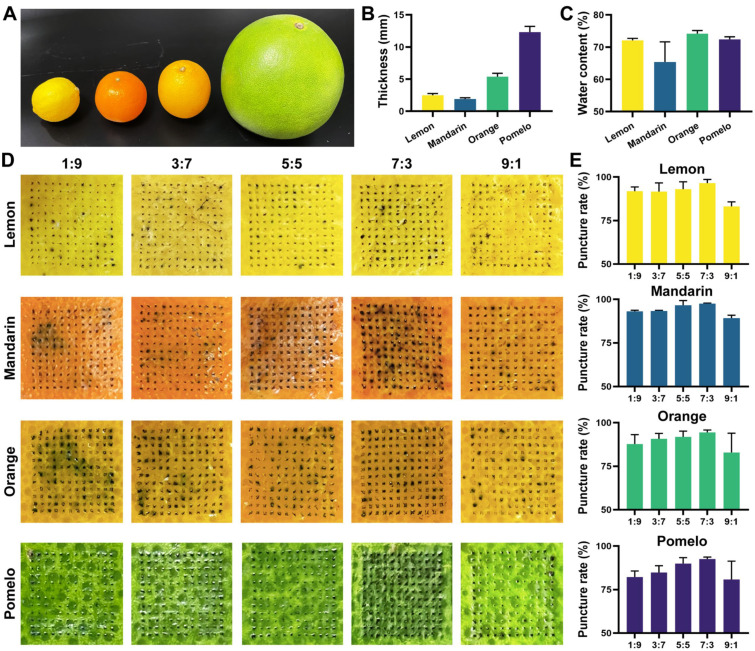
Characterization of pericarp insertion performance of different microneedles in citrus fruits. (**A**) Photographs of lemon, Satsuma mandarin, Valencia oranges and green pomelo. Water content (**B**) and pericarp thickness (**C**) of different fruits. (**D**) Photographs of the pericarp after the treatment of different microneedles and stained with Trypan blue. (**E**) Pericarp insertion rates of different microneedles in different fruits.

To determine the optimal ratio of ε-PL and PVA, microneedles with different ratios of ε-PL and PVA were prepared. Citrus fruits such as lemon, Satsuma mandarin, Valencia oranges, and green pomelo were selected for this study and characterized for their basic pericarp properties (Figure 2A–C). Because trypan blue can stain broken tissues [35], the pericarp insertion performance of different microneedles was evaluated using trypan blue staining (Figure 2D,E).

As shown in Figure 2D,E, different ratios of ε-PL and PVA had a greater impact on the pericarp insertion performance. At a 9:1 ratio between ε-PL and PVA, the microneedles with lower PVA content may be brittle, resulting in easy breakage of the microneedles during skin puncture. Moreover, owing to the high hygroscopicity of ε-PL, premature dissolution of microneedles during skin puncture may also significantly affect the insertion depth of the microneedles. Conversely, lower skin puncture at high-PVA-specific gravity may be due to the lack of sufficient stiffness of the microneedle to puncture the fruit pericarp.

Because the thickness of the pericarp is related to its mechanical properties, the moisture content of the pericarp also affects the dissolution rate of microneedles [36,37]. We compared the skin thickness and water content of four fruits: lemon, Satsuma mandarin, Valencia oranges, and green pomelo. The fruits with the thinnest and thickest pericarps included Satsuma mandarin and green pomelo, respectively. In addition, the fruits with the smallest and largest water content included Satsuma mandarin and Valencia oranges, respectively. However, the highest insertion rate of approximately 96% was obtained for microneedles with a 7:3 ratio of ε-PL to PVA in all the fruit puncture tests (Figure 2E). This result indicates that microneedles with a 7:3 ratio of ε-PL to PVA possess both good mechanical properties and resistance to moisture. Thus, we selected this formulation as the optimal formulation for the BMN construction and used it in the subsequent evaluation.

### 3.3. Characterization of BMN

**Figure 3:**
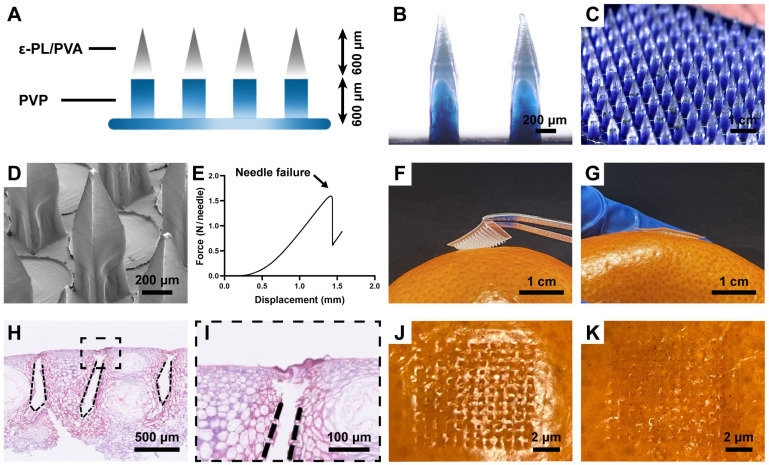
Structural characterization and insertion performance of BMN. (**A**) Schematic of the microneedle tip structure. Microscopic (**B**) and camera image (**C**) of BMN with the basal layer stained with trypan blue. (**D**) SEM image of BMN. (**E**) Failure behavior of BMN under axial load. Photograph of Satsuma mandarin before (**F**) and after BMN treatment (**G**). H&E-stained image of BMN after puncture into the pericarp of Satsuma mandarin (**H**) and its local magnification (**I**). Photograph of the pericarp of Satsuma mandarin before (**J**) and after 3 h of BMN insertion (**K**).

BMN is expected to have a well-defined structure, with ε-PL being enriched at the tip of the needle (Figure 3A). Therefore, we stained the PVP-based substrate layer with trypan blue. As shown in Figure 3B,C, the microneedle tip of ε-PL and PVA hybridization had an intact needle shape, no air bubbles at the tip, and a clear partitioning between the ε-PL-containing layer of the tip and the substrate layer. The ε-PL-containing tip layer was distributed in the region 0~600 μm from the tip. SEM images of this microneedle were obtained to observe the microstructure of the BMNs. Figure 3D shows that the BMN tip was intact with no obvious fine defects, indicating that ε-PL is compatible with PVA.

We quantified the mechanical properties of the needle tip of the BMN using a texture analyzer and measured the force that a microneedle could withstand before failure using the method of Park et al. [36]. Stress versus strain curves of the BMN were thus obtained, and the maximum force applied immediately before dropping was identified as the force of needle failure. As shown in Figure 3E, the prepared BMN microneedles showed a failure force of 1.6 N per needle, indicating that the microneedles should have sufficient strength to penetrate the pericarp without breaking.

To further investigate the insertion performance of BMN into the pericarp, we collected Satsuma mandarin pericarps after microneedle administration for H&E staining (Figure 3F,G). Figure 3H,I shows that BMN could form distinct micropore channels in the Satsuma mandarin pericarp after puncture, reaching depths between 660 and 920 μm. Notably, microneedle systems usually penetrate mammalian skin with a penetration depth of merely 50–400 μm, because the skin surface is more deformable than fruit pericarps [17,18,19,20,21]. The deeper micropore channels caused by the microneedle penetration into the pericarp imply that ε-PL could be delivered to deeper tissues via BMN, which also illustrated the great application potential of the microneedle system in protecting fruits from postharvest infection.

Interestingly, we found that these micropore channels would naturally close after BMN administration, probably because of tissue wrinkling caused by water evaporation from the pericarp surface (Figure 3I). The hole on the pericarp surface gradually became less visible within 3 h (Figure 3J,K), which may have resulted in an improved appearance of the fruit and hindered the invasion of external microorganisms.

### 3.4. Dissolution Properties of BMN in Fruit Pericarp

**Figure 4:**
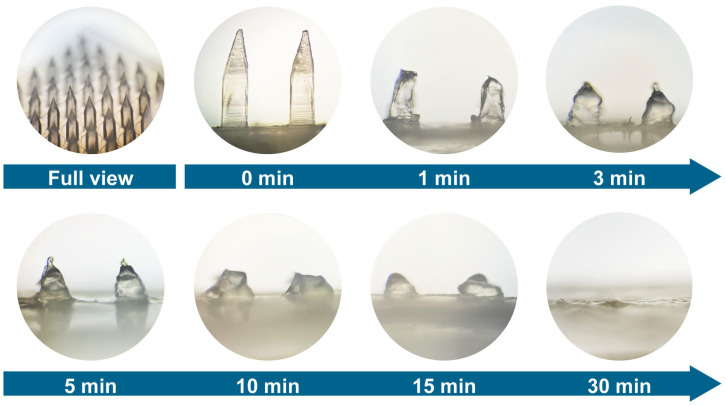
Appearance of BMN and its needle tip length after insertion into citrus pericarp for different times.

To evaluate the dissolution behavior and dissolution rate of BMN after puncturing the pericarp, the microneedle was observed by microscopy after puncturing the citrus skin at different times. As shown in Figure 4, the ε-PL-containing needle tip dissolved completely after 3 min of BMN piercing the pericarp, and the entire microneedle tip disappeared completely within 30 min. This experiment indicated that the needle tips of BMN have good solubility and that BMN could promote the ε-PL efficiently pass through the outer cuticles of fruit pericarp.

### 3.5. Antifungal Potential of BMN

As a natural antimicrobial peptide, ε-PL usually kills pathogens through a membrane disruption mechanism [38,39]. While the fungal cell membrane is negatively charged, cationic ε-PL can electrostatically bind to cells with little dependence on specific receptors or essential components of the fungal cell membranes [40]. Therefore, various previous studies have indicated that ε-PL displays effective antifungal activity against a range of plant pathogenic fungi, which may result in cell dysfunction and suppression of spore germination or mycelial growth [41,42,43,44,45,46]. ε-PL can inhibit common postharvest pathogens, including *Alternaria alternata*, *Botrytis cinerea*, *Penicillium expansum*, and *Penicillium digitatum*, with half-maximal inhibitory concentration (IC_50_) values of 30–200 μg/mL [46].

To investigate the antifungal potential of BMN, we separated the needle tips of BMN and measured the ε-PL content using HPLC. We found that the drug-loading capacity of BMN was very high, and the microneedle patch contained up to 1890 μg of ε-PL per patch. As ε-PL could suppress fungal cell viability in a concentration-dependent manner, a higher ε-PL loading capacity implies that BMN may be more effective in protecting fruits from postharvest infection.

### 3.6. Drug Distribution Study

**Figure 5:**
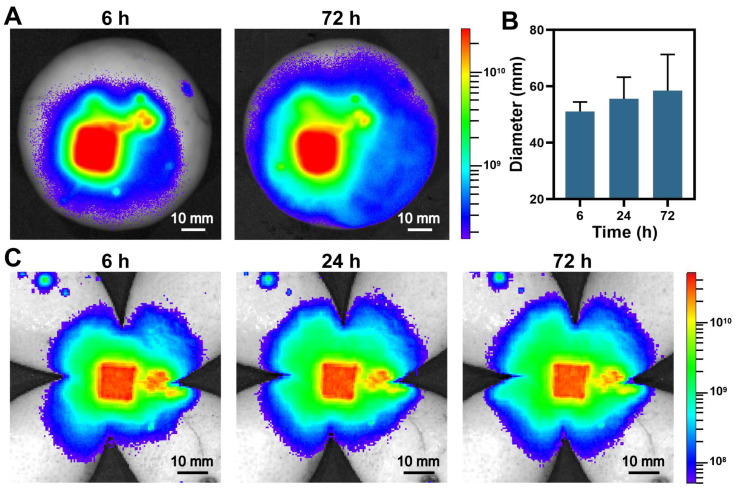
Characterization of ε-PL distribution after BMN administration. (**A**) Representative fluorescence images of citrus after Cy7-labeled BMN administration. The diameter changes (**B**) and representative images (**C**) of the fluorescent area on the pericarp of citrus after Cy7-labeled BMN administration.

Microneedle patches have been widely applied in the treatment of animal diseases, and drugs can be rapidly and systematically distributed through the blood circulation. When microneedles are used to fruit preservatives, drug distribution may be achieved mainly through concentration gradient-mediated drug diffusion. Therefore, to evaluate the unique mode of action of microneedles for topical application in fruits, we constructed a BMN using Cy7-labeled ε-PL, and administered them to the surface of intact citrus and isolated pericarp, respectively. Interestingly, we found that the diffusion of ε-PL was rapid within 6 h of microneedle administration, and the distribution area of ε-PL remained increased over a period of 72 h (Figure 5A). To quantify the distribution of ε-PL on the pericarp, we further isolated the pericarp of citrus, administered microneedles in the center of the pericarp, and measured the diameter of fluorescent area. As shown in Figure 5B,C, a large concentration difference facilitated the rapid diffusion of ε-PL from BMN to the pericarp forming a fluorescent area with a diameter of 51.1 ± 3.3 mm at 6 h post-BMN administration, and this area could was continuously enlarged to 58 ± 12.8 mm in diameter after 72 h. These results indicated that although distributed primarily by diffusion, BMN can deliver ε-PL to the pericarp and create a large drug distribution area, and BMN has great potential to reduce the incidence of invasive fungal infections in local areas of citrus fruit pericarp (e.g., areas with localized damage).

### 3.7. Cytocompatibility Study

**Figure 6:**
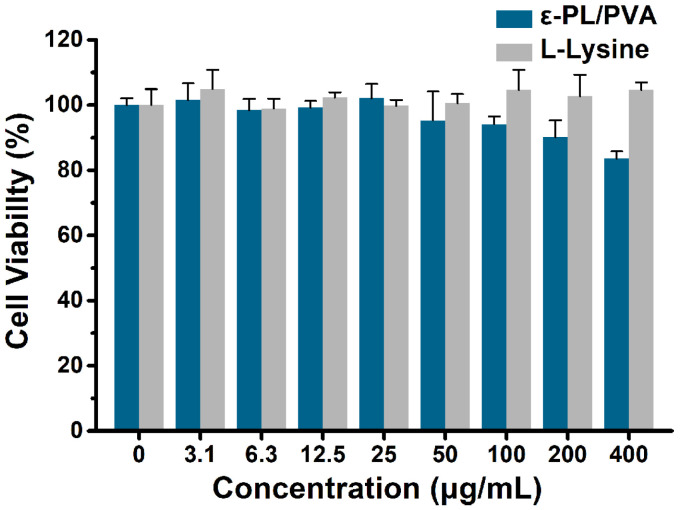
Cell viability of NCM460 cells after treatment with different concentrations of ε-PL/PVA mixture or L-lysine hydrochloride.

To investigate whether the tip material of the BMN could induce potential toxicity to cells in the gastrointestinal tract, the CCK-8 method was used to explore the cytocompatibility of the mixture of ε-PL and PVA (7:3) [47]. Figure 6 demonstrates that the mixture of ε-PL and PVA showed little cytotoxicity against NCM460 cells.

As lysine-based ε-PL can be degraded by enzymes in the gastrointestinal tract, we further investigated the effect of different concentrations of L-lysine hydrochloride on the viability of NCM460 cells. As shown in Figure 6, the degradation products of ε-PL had little effect on the survival of cells in the gastrointestinal tract, which further suggests that BMN needle-tip excipients may be unlikely to cause significant toxic effects after consumption.

### 3.8. Adhesive Outer Layer and Its Potential Applications

**Figure 7:**
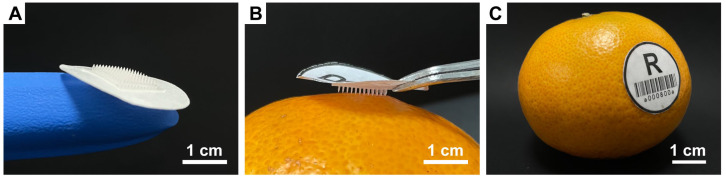
Appearance of the adhesive outer layer of BMN (**A**) and the photograph of Satsuma mandarin before (**B**) and after the microneedle patches treatment (**C**).

To prevent BMN from falling off during use and to increase the sealing of holes created by the microneedles, an additional layer consisting of an adhesive sticker can be added to the outside of the PVP layer of the BMN. As shown in Figure 7, with the addition of the adhesive outer layer, BMN can be firmly attached to the surface of citrus. By printing text and/or pattern on the surface of the adhesive outer layer, BMN can also be used to provide information on the fruit’s production time, preservation conditions, price, and other product information.

## 4. Conclusions

In this study, the concept of biocontrol microneedles was proposed to develop a novel biocontrol agent for eliminating postharvest fungal pathogens by exploiting the high drug transdermal delivery properties of the dissolving microneedle system. We successfully optimized the feeding ratio of ε-PL to PVA for the construction of a biocontrol microneedle at 7:3 through an ex vivo insertion test of various microneedles in citrus fruit pericarps. Moreover, BMN was confirmed to have excellent mechanical properties and could be inserted into the pericarp of different citrus fruits with an approximately 96% insertion rate. After insertion into the pericarp, ε-PL located at the tip of the microneedle can be rapidly dissolved and released into the pericarp within 3 min. This microneedle has a high ε-PL loading capacity, which allows a concentration-dependent antimicrobial agent such as ε-PL to exert greater antimicrobial efficacy. The drug distribution study showed that ε-PL could be diffused from the local area after microneedle administration. Moreover, CCK-8 experiments verified the low cytotoxicity of ε-PL and PVA mixtures and the degradation products of ε-PL. Therefore, this study preliminarily confirmed the feasibility and application potential of BMN, and it is believed that it can be applied to protect more types of fruits or vegetables from postharvest infection through further optimization. Notably, we calculated that the raw material cost to produce a BMN is no more than USD 0.005, but the main and high cost of fabricating BMN may be attributed to the manufacturing process. Given that the cost of manufacturing remains a significant uncertainty, robust and cost-effective manufacturing of the BMN may be another important issue needing further study and development.

## Data Availability

Data are available on request.
